# ULTRA: Universal Grammar as a Universal Parser

**DOI:** 10.3389/fpsyg.2018.00155

**Published:** 2018-02-15

**Authors:** David P. Medeiros

**Affiliations:** Department of Linguistics, University of Arizona, Tucson, AZ, United States

**Keywords:** syntax, linearization, parsing, universal grammar, word order, typology, universal 20, stack-sorting

## Abstract

A central concern of generative grammar is the relationship between hierarchy and word order, traditionally understood as two dimensions of a single syntactic representation. A related concern is directionality in the grammar. Traditional approaches posit process-neutral grammars, embodying knowledge of language, put to use with infinite facility both for production and comprehension. This has crystallized in the view of Merge as the central property of syntax, perhaps its only novel feature. A growing number of approaches explore grammars with different directionalities, often with more direct connections to performance mechanisms. This paper describes a novel model of universal grammar as a one-directional, universal parser. Mismatch between word order and interpretation order is pervasive in comprehension; in the present model, word order is language-particular and interpretation order (i.e., hierarchy) is universal. These orders are not two dimensions of a unified abstract object (e.g., precedence and dominance in a single tree); rather, both are temporal sequences, and UG is an invariant real-time procedure (based on Knuth's stack-sorting algorithm) transforming word order into hierarchical order. This shift in perspective has several desirable consequences. It collapses linearization, displacement, and composition into a single performance process. The architecture provides a novel source of brackets (labeled unambiguously and without search), which are understood not as part-whole constituency relations, but as storage and retrieval routines in parsing. It also explains why neutral word order within single syntactic cycles avoids 213-like permutations. The model identifies cycles as extended projections of lexical heads, grounding the notion of phase. This is achieved with a universal processor, dispensing with parameters. The empirical focus is word order in noun phrases. This domain provides some of the clearest evidence for 213-avoidance as a cross-linguistic word order generalization. Importantly, recursive phrase structure “bottoms out” in noun phrases, which are typically a single cycle (though further cycles may be embedded, e.g., relative clauses). By contrast, a simple transitive clause plausibly involves two cycles (vP and CP), embedding further nominal cycles. In the present theory, recursion is fundamentally distinct from structure-building within a single cycle, and different word order restrictions might emerge in larger domains like clauses.

## Introduction

One of the most significant recent developments for linguistic theory is the appearance of high-quality datasets on the full range of cross-linguistic variation. In the past, generative studies typically relied on detailed examination of one or several languages to illuminate syntactic mechanisms. While this approach is certainly fruitful, the accumulation of information about large numbers of languages opens new possibilities for sharpening understanding.

Within generative grammar, considerable attention has been given to recursion as a (or even *the*) fundamental property of language (see Berwick and Chomsky, 2016 for discussion). This is formalized in a core operation called Merge, combining two syntactic objects (ultimately built from lexical items) into a set containing both. Recursion follows from the ability of Merge to apply to its own output. Merge also captures the essential fact that sentences have internal structure (bracketed constituency), each layer corresponding to an application of Merge.

Contrary to this framework, I argue that it is a conceptual error to view sentences as groupings (whether sets, or something else) of lexical items. The error inheres in thinking of lexical items as coherent units existing at a single level. This leads to thinking of sentences as single-level representations as well. Words, put simply, aren't *things*; they are a pair of processes, extended in time. In the context of comprehension, the relevant processes are recognition of the word, and integration of its meaning into an interpretation. I develop a novel view of the structure of sentences in terms of these two kinds of processes. Crucially, a non-trivial relationship governs their relative sequencing: one word may occur earlier than another in surface order, yet its meaning may be integrated later. Considering sentences as unified, atemporal representations built atop impenetrable lexical atoms leaves us unable to capture the fundamentally temporal phenomena involved, in which the two aspects of each word are not bundled together, and the processes for different words interweave.

This paper proposes a novel model of grammatical mechanisms, called ULTRA (Universal Linear Transduction Reactive Automaton). Within local syntactic domains forming the extended projection of a lexical root (such as a verb or noun), ULTRA employs Knuth's (1968) stack-sorting algorithm to directly map surface word orders to underlying base structure. The mapping succeeds only for 213-avoiding orders. This is an intriguing result, as 213-avoidance arguably bounds neutral word order variation across languages, in a variety of syntactic domains. While the local sound and meaning representations in this model are sequences, hierarchical structure nevertheless arises in the dynamic action of the mapping. The bracketed structures found here, although epiphenomenal, closely match those built by Merge, with some crucial differences (arguably favoring the present theory).

Stack-sorting proves to be an effective procedure for linking word order and hierarchical interpretation, encompassing linearization, displacement, composition, and labeled brackets. The theory invites realization as a real-time performance process. Pursuing that realization significantly recasts the boundaries between performance and competence. Remarkably, ULTRA requires no language-particular parameters; an invariant algorithm serves as grammatical device for all languages. Put simply, I propose that Universal Grammar is a universal parser.

Nevertheless, stack-sorting is too limited a mechanism to describe all the phenomena of human syntax. Three kinds of effects are left hanging: unbounded recursion, non-neutral orders, and the existence of apparently distinct languages. Moreover, understanding stack-sorting as a processing system encounters two obvious problems: it is a unidirectional parser, not trivially reversible for production; and it conflicts with strong evidence for word-by-word incrementality in comprehension.

Although constructing a complete model of syntax and processing goes far beyond the scope of the paper, the problems that arise in basing a parser-as-grammar model on stack-sorting warrant consideration. I appeal to the distinction between reactive and predictive processes, casting stack-sorting as a universal reactive routine. A separate predictive module plays a crucial role in production, and in the appearance of distinct, relatively rigid word orders. Prediction also helps reconcile ULTRA with incremental interpretation. I appeal to properties of memory to resolve further problems, speculating that primacy memory (distinct from the recency memory underpinning stack-sorting) is the source of another cluster of syntactic properties, including long-distance movement, crossing dependencies, and the special syntax of the “left periphery.” Finally, I suggest that episodic memory—independently hierarchical in structure, in humans—plays a key role in linguistic recursion.

The structure of this paper is as follows. Section Linear Base argues that the “base” structure within each local syntactic domain is a sequence. Section ^*^213 in Neutral Word Order explores the generalization that 213-avoidance delimits information-neutral word order possibilities, across languages. Section Stack-Sorting as a Grammatical Mechanism proposes a stack-sorting procedure to capture 213-avoidance in word order. Section Stack-Sorting: Linearization, Displacement, Composition, and Labeled Brackets shows how further syntactic effects follow from stack-sorting. Section Comparison with Existing Accounts of Universal 20 compares ULTRA to existing accounts of 213-avoidance in word order, focusing on Universal 20. Section Universal Grammar as Universal Parser pursues the realization of stack-sorting in real-time performance. Section Possible Extensions to a More Complete Theory of Syntax addresses the challenges in taking stack-sorting as the core of Universal Grammar, sketching some possible extensions. Section Conclusion concludes.

## Linear base

Syntactic combination could take many forms. An emerging view is that combination largely keeps to head-complement relations (Starke, 2004; Jayaseelan, 2008). The term “head” has at least two different senses, in this context. First, in any combination of two syntactic objects, one is “more central” to the composite meaning. Let us call this notion of head the *root*, noting that in extended projections of nouns and verbs, the lexical noun or verb root is semantically dominant. The other sense of head concerns which element determines the combinatoric behavior of the composite; let us call this notion of head the *label*.

In older theories of phrase structure, the two senses of head (root and label) converged on the same element; a noun, for example, combined with all its modifiers within a noun phrase. Headedness thus mapped to hierarchical dominance; the root projected its label above its dependents. To illustrate, a combination of adjective and noun, such as *red books*, would be represented as follows.

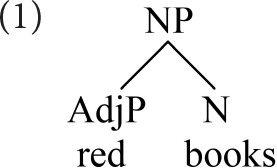

This traditional conclusion about the relationship of dependency and hierarchy is overturned in modern syntactic cartography (Rizzi, 1997; Cinque, 1999, and subsequent work). Cartographic approaches propose that syntactic combination follows a strict, cross-linguistically uniform hierarchy, within each extended projection. This hierarchy involves a sequence of functional heads, licensing combination with various modifiers in rigid order. The phrase *red books* is represented as follows.

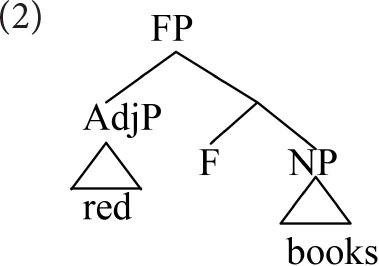

Here, the adjective is the specifier of a dedicated functional head (F), which labels the composite, determining its combinatoric behavior. In cartographic representations heads are uniformly *below* their dependents, which appear higher up the spine.

Questions arise about these representations, which postulate an abundance of unpronounced material. A curious observation is that functional heads and their specifiers seem not to occur together overtly, as formalized in Koopman's (2000) Generalized Doubly-Filled Comp Filter.

Starke (2004) takes Koopman's observation further, arguing that heads and specifiers do not co-occur because they are *tokens of the same type*, competing for a single position. Starke recasts the cartographic spine as an abstract functional sequence (*fseq*), whose positions can be discharged equally by lexical or phrasal material. Pursuing Starke's conception, the adjective-noun combination would be represented as below.

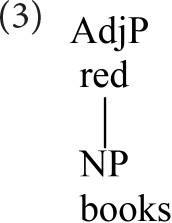

Again, we have reversed traditional conclusions about the hierarchy of heads and dependents. Nevertheless, the notion of root (picking out the noun) is still crucial, as the modifiers occur in the hierarchical order dictated by *its* fseq.

Syntactic combination of this sort is sequential, within each extended projection. These “base” sequences encode bottom-up composition, so it is natural to order the sequence in the same way (bottom-up). The base (i.e., fseq, cartographic spine) is widely taken to be uniform across languages, and to express “thematic,” information-neutral meaning (contrasted with discourse-information structure)^1^.

A grammar, on anyone's theory, specifies a formal mapping linking sound and meaning (more accurately, outer and inner form, allowing for non-auditory modalities). This specification could take many forms. Sequential representation of the base allows a remarkably simple formulation of the sound-meaning mapping. This reformulation yields a principled account of a class of word order universals. Moreover, while the interface objects (word orders, and base trees) involved in the mapping are sequences, bracketed hierarchical structure arises as a dynamic effect.

There are various ways of conceptualizing the relationship between the base and surface word order. The usual view is that the base orders the input to a derivation, yielding surface word order as the output. That directionality is implicit in terms used to describe the hierarchy-order relation: *linearization, externalization*, etc. This paper pursues a different view, where surface word orders are inputs to an algorithm that attempts to assemble the base as output. Significantly, the only inputs that converge on the uniform base under this process are 213-avoiding; all 213-containing word orders result in deviant output.

## ^*^213 In neutral word order

213-avoidance arguably captures information-neutral word order possibilities in a variety of syntactic domains, across languages. By 213-avoidance, I mean a ban on surface order …*b…a…c*…, for elements *a* ≫ *b* ≫ *c*, where ≫ indicates c-command in standard tree representations of the base (equivalently, dominance in Starke's trees). In other words, neutral word orders seem to avoid a mid-high-low (sub)sequence of elements from a single fseq. The elements forming this forbidden contour need not be adjacent, in surface order or in the base fseq.

213-avoidance is widely believed to delimit the ordering options for verb clusters, well-known in West Germanic (see Wurmbrand, 2006 for an overview). Barbiers et al.'s (2008) extensive survey of Dutch dialects found very few instances of this order; German dialects seem to avoid this order as well^2^. Meanwhile Zwart (2007) analyzes 213 order in Dutch verb clusters as involving extraposition of the final element^3^.

The best-studied domain supporting 213-avoidance in word order is Greenberg's Universal 20, describing noun phrase orders.

“When any or all of the items (demonstrative, numeral, and descriptive adjective) precede the noun, they are always found in that order. If they follow, the order is either the same or its exact opposite” (Greenberg, 1963 p. 87).

Subsequent work has refined this picture. Cinque (2005) reports that only 14 of 24 logically possible orders of these elements are attested as information-neutral orders (Table 1).

**Table 1:** Possible noun phrase orders. Cinque (2005, pp. 319–320) report of the number of languages exhibiting each order is given by a number: 0 = unattested; 1 = very few languages; 2 = few languages; 3 = many languages; 4 = very many languages.

Dem Num Adj N 4	Dem Num N Adj 3	Dem N Num Adj 1	N Dem Num Adj 2
^*^Num Dem Adj N 0	^*^Num Dem N Adj 0	^*^Num N Dem Adj 0	^*^N Num Dem Adj 0
^*^Adj Dem Num N 0	^*^Adj Dem N Num 0	Adj N Dem Num 1	N Adj Dem Num 2
^*^Dem Adj Num N 0	Dem Adj N Num 1	Dem N Adj Num 3	N Dem Adj Num 1
^*^Num Adj Dem N 0	Num Adj N Dem 1	Num N Adj Dem 2	N Num Adj Dem 2
^*^Adj Num Dem N 0	^*^Adj Num N Dem 0	Adj N Num Dem 1	N Adj Num Dem 4

*Cells with unattested orders are shaded for additional clarity. Attested orders are all and only the 213-avoiding permutations of the Dem ≫ Adj ≫ Num ≫ N base*.

Cinque describes these facts with a constraint on movement from a uniform base. Specifically, he proposes that all movements move the noun, or something containing it, to the left (section Comparison with Existing Accounts of Universal 20 details Cinque's theory and related accounts). What is forbidden is remnant movement.

Noun phrase orders obey a simple generalization: attested orders are all and only 213-avoiding permutations. All unattested orders have 213-like subsequences. For example, unattested ^*^Num Dem Adj N contains subsequences *Num Dem Adj*…, and *Num Dem … N*, representing mid-high-low contours with respect to the fseq.

## Stack-sorting as a grammatical mechanism

There is a particularly simple procedure that maps 213-avoiding word^4^ orders to the uniform base, called stack-sorting^5^. I describe an adaptation of Knuth (1968) stack-sorting algorithm, which uses last-in, first out (stack) memory to sort items by their relative order in the base. This is a partial sorting algorithm: it only achieves the desired output for some input orders.

**Table:** 

(4)	Stack-sorting algorithm	Definitions
	While input is non-empty,	I: next item in input.
	If I ≫ S, Pop.	S: item on top of stack.
	Else Push.	x ≫ y: x c-commands y in the base (e.g., Dem ≫ N).
	While Stack is non-empty,	Push: moves I from input onto stack.
	Pop.	Pop: moves S from stack to output.

(4) maps all and only 213-avoiding word orders to a 321-like hierarchy, corresponding to the base. 213-containing orders are mapped to a deviant output, distinct from the base. By hypothesis, that is why such orders are typologically unavailable: they are automatically mapped to an uninterpretable order of composition. This explains the Universal 20 pattern (Tables 2, 3).

**Table 2:** Result of stack-sorting logically possible orders of 4 elements, in the format **input** → output.

**1234** → 4321	**1243** → 4321	**1423** → 4321	**4123** → 4321
^*^2134 → ^*^2431	^*^2143 → ^*^2431	^*^2413 → ^*^4231	^*^4213 → ^*^4231
^*^3124 → ^*^3421	^*^3142 → ^*^3421	**3412** → 4321	**4312** → 4321
^*^1324 → ^*^3421	**1342** → 4321	**1432** → 4321	**4132** → 4321
^*^2314 → ^*^3241	**2341** → 4321	**2431** → 4321	**4231** → 4321
^*^3214 → ^*^3241	^*^3241 → ^*^3421	**3421** → 4321	**4321** → 4321

*213-avoiding orders (white cells) are stack-sorted into the 4,321 base sequence. Note that the correctly stack-sorted orders correspond exactly to the attested noun phrase orders, as reported by Cinque (2005)*.

**Table 3:** Stack sorting computations for 4-orders.

	**Output ← Stack ← Input**				**Output ← Stack ← Input**				**Output ← Stack ← Input**				**Output ← Stack ← Input**		
Start	-	-	**1234**	Start	-	-	**1243**	Start	-	-	**1423**	Start	-	-	**4123**
Push 1		1	234	Push 1		1	243	Push 1		1	423	Push 4		4	123
Push 2		2/1	34	Push 2		2/1	43	Push 4		4/1	23	Pop 4	4		123
Push 3		3/2/1	4	Push 4		4/2/1	3	Pop 4	4	2/1	23	Push 1	4	1	23
Push 4		4/3/2/1		Pop 4	4	2/1	3	Push 2	4	2/1	3	Push 2	4	2/1	3
Pop 4	4	3/2/1		Push 3	4	3/2/1		Push 3	4	3/2/1		Push 3	4	3/2/1	
Pop 3	43	2/1		Pop 3	43	2/1		Pop 3	43	2/1		Pop 3	43	2/1	
Pop 2	432	1		Pop 2	432	1		Pop 2	432	1		Pop 2	432	1	
Pop 1	**4321**			Pop 1	**4321**			Pop 1	**4321**			Pop 1	**4321**		
	**Output ← Stack ← Input**				**Output ← Stack ← Input**				**Output ← Stack ← Input**				**Output ← Stack ← Input**		
Start	-	-	^*^2134	Start	-	-	^*^2143	Start	-	-	^*^2413	Start	-	-	^*^4213
Push 2		2	134	Push 2		2	143	Push 2		2	413	Push 4		4	213
Pop 2	2		134	Pop 2	2		143	Push 4		4/2	13	Pop 4	4		213
Push 1	2	1	34	Push 1	2	1	43	Pop 4	4	2	13	Push 2	4	2	13
Push 3	2	3/1	4	Push 4	2	4/1	3	Pop 2	42		13	Pop 2	42		13
Push 4	2	4/3/1		Pop 4	24	1	3	Push 1	42	1	3	Push 1	42	1	3
Pop 4	24	3/1		Push 3	24	3/1		Push 3	42	3/1		Push 3	42	3/1	
Pop 3	243	1		Pop 3	243	1		Pop 3	423	1		Pop 3	423	1	
Pop 1	^*^2431			Pop 1	^*^2431			Pop 1	^*^4231			Pop 1	^*^4231		
	**Output ← Stack ← Input**				**Output ← Stack ← Input**				**Output ← Stack ← Input**				**Output ← Stack ← Input**		
Start	-	-	^*^3124	Start	**-**	-	^*^3142	Start	**-**	-	**3412**	Start	-	-	**4312**
Push 3		3	124	Push 3		3	142	Push 3		3	412	Push 4		4	312
Pop 3	3		124	Pop 3	3		142	Push 4		4/3	12	Pop 4	4		312
Push 1	3	1	24	Push 1	3	1	42	Pop 4	4	3	12	Push 3	4	3	12
Push 2	3	2/1	4	Push 4	3	4/1	2	Pop 3	43		12	Pop 3	43		12
Push 4	3	4/2/1		Pop 4	34	1	2	Push 1	43	1	2	Push 1	43		1
Pop 4	34	2/1		Push 2	34	2/1		Push 2	43	2/1		Push 2	43	2/1	
Pop 2	342	1		Pop 2	342	1		Pop2	432	1		Pop 2	432	1	
Pop 1	^*^3421			Pop 1	^*^3421			Pop 1	**4321**			Pop 1	**4321**		
	**Output ← Stack ← Input**				**Output ← Stack ← Input**				**Output ← Stack ← Input**				**Output ← Stack ← Input**		
Start	-	-	^*^1324	Start	-	-	**1342**	Start	-	-	**1432**	Start	-	-	**4231**
Push 1		1	324	Push 1		1	342	Push 1		1	431	Push 4		4	132
Push 3		3/1	24	Push 3		3/1	42	Push 4		4/1	32	Pop 4	4		132
Pop 3	3	1	24	Push 4		4/3/1	2	Pop 4	4	1	32	Push 1	4	1	32
Push 2	3	2/1	4	Pop 4	4	3/1	2	Push 3	4	3/1	2	Push 3	4	3/1	2
Push 4	3	4/2/1		Pop 3	43	1	2	Pop 3	43	1	2	Pop 3	43	2/1	2
Pop 4	34	2/1		Push 2	43	2/1		Push 2	432	2/1		Push 2	43	1	
Pop 2	342	1		Pop 2	432	1		Pop 2	432	1		Pop 2	432		
Pop 1	^*^3421			Pop 1	**4321**			Pop 1	**4321**			Pop 1	**4321**		
	**Output ← Stack ← Input**				**Output ← Stack ← Input**				**Output ← Stack ← Input**				**Output ← Stack ← Input**		
Start	-	-	^*^2314	Start	-	-	**2341**	Start	-	-	**2431**	Start			**4321**
Push 2		2	314	Push 2		2	341	Push 2		2	431	Push 4		4	231
Push 3		3/2	14	Push 3		3/2	41	Push 4		4/2	31	Pop 4	4		231
Pop 3	3	2	14	Push 4		4/3/2	1	Pop 4	4	2	31	Push 2	4	2	31
Pop 2	32		14	Pop 4	4	3/2	1	Push 3	4	3/2	1	Push 3	4	3/2	1
Push 1	32	1	4	Pop 3	43	2	1	Pop 3	43	2	1	Pop 3	43	2	1
Push 4	32	4/1		Pop 2	432		1	Pop 2	432		1	Pop 2	432		1
Pop 4	324	1		Push 1	432	1		Push 1	432	1		Push 1	432	1	
Pop 1	^*^3241			Pop 1	**4321**			Pop 1	**4321**			Pop 1	**4321**		
	**Output ← Stack ← Input**				**Output ← Stack ← Input**				**Output ← Stack ← Input**				**Output ← Stack ← Input**		
Start	-	-	^*^3214	Start	-	-	^*^3214	Start	-	-	**3421**	Start			**4321**
Push 3		3	214	Push 3		3	241	Push 3		3	421	Push 4		4	321
Pop 3	3		214	Pop 3	3		241	Push 4		4/3	21	Pop 4	4		321
Push 2	3	2	14	Push 2	3	2	41	Pop 4	4	3	21	Push 3	4	3	21
Pop 2	32		14	Push 4	3	4/2	1	Pop 3	43		21	Pop 3	43		21
Push 1	32	1	4	Pop 4	34	2	1	Push 2	43	2	1	Push 2	43	2	1
Push 4	32	4/1		Pop 2	342		1	Pop 2	432		1	Pop 2	432		1
Pop 4	324	1		Push 1	342	1		Push 1	432	1		Push 1	432	1	
Pop 1	^*^3241			Pop 1	^*^3421			Pop 1	**4321**			Pop 1	**4321**		

*All and only the 213-avoiding orders, corresponding to attested DP orders (Cinque, 2005), are sorted into 4321. Input word order (top right) and output interpretation order (bottom left) are in bold*.

Let us illustrate how (4) parses some noun phrase orders: Dem-Adj-N, N-Dem-Adj, ^*^Adj-Dem-Num.

(5) Dem-Adj-N: PUSH(Dem), PUSH(Adj), PUSH(N), POP(N), POP(Adj), POP(Dem).
(6) N-Dem-Adj: PUSH(N), POP(N), PUSH(Dem), PUSH(Adj), POP(Adj), POP(Dem).

For attested orders, the nominal categories POP in the order < N, Adj, Dem>, matching their bottom-up hierarchy.

(7) ^*^Adj-Dem-N: PUSH(Adj), POP(Adj), PUSH(Dem), PUSH(N), POP(N), POP(Dem).

For the unattested 213-like order, items POP in the deviant order ^*^ < Adj, N, Dem>, failing to construct the universal interpretation order.

That's nice: (4) maps attested orders to their universal meaning, simultaneously ruling out unattested orders. But beyond such a mapping, an adequate grammar must explain other aspects of knowledge of language, including surface structure bracketing. If grammar treats surface orders and base structures as sequences^6^ (locally), where can such bracketed structure come from?

## Stack-sorting: linearization, displacement, composition, and labeled brackets

In this section, I show that stack-sorting effectively encompasses linearization, displacement, and composition, as well as assigning brackets, labeled unambiguously and without search. Moreover, it does all of this without language-particular parameters.

In the standard (“Y-model”) view, linearization and composition are distinct interface operations, interpreting structures built in an autonomous syntactic module by Merge. In ULTRA, linearization goes in the other direction, loading surface word order item-by-item into memory, and reassembling it in order of compositional interpretation.

### Displacement is a natural property of a stack-sorting grammar

Displacement is a natural feature of stack-sorting; from one point of view, it is the basic property of the system. In standard accounts, constituents that compose together in the interpretation should appear adjacent in surface order. This arrangement is forced by phrase structure grammars. Displacement, whereby elements that compose together are separated by intervening elements in surface order, has always seemed a surprising property, in need of explanation.

Things work quite differently in ULTRA. A key assumption of the Merge-based view is discarded: there is no level of representation encompassing word order and the fseq within a unified higher-order object. Instead, word order and base hierarchy are disconnected sequences, related dynamically. Non-adjacent input elements can perfectly well end up adjacent in the output. Displacement, rather than being the exception, is the rule; *every* element in the surface order is “transformed,” passing through memory before retrieval for interpretation^7^.

### Brackets and labels without primitive constituency

The algorithm (4) implicitly assigns labeled bracketed structure^8^ to each surface order, matching almost exactly the structures assigned by accounts like Cinque (2005). Explicitly, pushing (storage from word order to stack) corresponds to a left bracket, and popping (retrieval from stack for interpretation) to a right bracket. These operations apply to one element at a time; it is natural to think of that element as labeling the relevant bracket. See Table 4, which provides the stack-sorting computations for all surface permutations of a 3-element base.

**Table 4:** Stack-sorting computations for orders of 3 elements.

	**Output**	**←**	**Stack**	**←**	**Input**		**Output**	**←**	**Stack**	**←**	**Input**
Start	-		-		123	Start	-		-		231
[ Push			1		23	[ Push		2	31		
[ Push			2/1		3	[ Push		3/2	1		
[ Push			3/2/1			[ Pop	3	2	1		
] Pop	3		2/1			] Pop	32		1		
] Pop	32		1			] Pop	32	1			
] Pop	**321**					] Pop	**321**				
	**Output**	**←**	**Stack**	**←**	**Input**		**Output**	**←**	**Stack**	**←**	**Input**
Start	-		-		**132**	Start	-		-		**321**
[ Push			1		32	[ Push			3		12
[ Push			3/1		2	] Pop	3				12
[ Pop	3		1		2	[ Push	3		1		2
] Pop	3		2/1		1	[ Push	3		2/1		
[ Push	32				1	] Pop	32				
] Pop	**321**					] Pop	**321**				
	**Output**	**←**	**Stack**	**←**	**Input**		**Output**	**←**	**Stack**	**←**	**Input**
Start	-		-		**213**	Start	-		-		**312**
[ Push			2		13	[ Push			3		21
] Pop	2				13	] Pop	3				21
[ Push	2		1		3	[ Push	3		2		1
[ Push	2		3/1			] Pop	32				1
] Pop	23		1			[ Push	32		1		
] Pop	*231		**FAILED SORT**			] Pop	**321**				

*Each order induces a unique sequence of pushes and pops, annotated with left or right brackets, respectively. The surface order is at topright within each computation, passing sequentially though memory to the output, at bottom left*.

**Figure 1:**
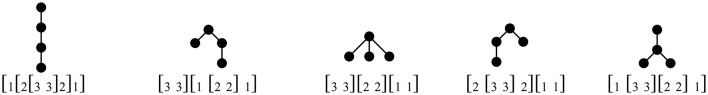
Brackets, and corresponding push-pop trees, for accepted (stack-sortable) orders of three elements. These are simply the Dyck trees with 4 nodes.

**Figure 2:**
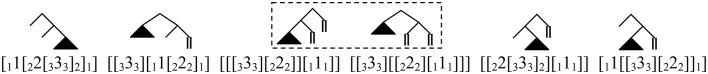
Binary-branching trees for remnant-movement-avoiding derivations of attested orders of three elements, with corresponding bracketing. The lexical root (e.g., N in a noun phrase) is shown as a black triangle, while structures with a terminal and trace of movement are represented with a double branch ||. The trees are represented this way to highlight the correspondence with the Dyck trees for these orders derived from stack-sorting.

Examining these brackets, the sequence of pushes and pops (storage and retrieval) for each order implicitly defines a tree, as shown in Figure 1. These are the so-called Dyck trees^9^, the set of all ordered rooted trees with a fixed number of nodes (here, 4). Compare these to the binary-branching trees assigned under Cinque's (2005) account, with non-remnant, leftward movement affecting a right-branching base (Figure 2). The brackets are nearly identical, as are their labels, taking some liberties with the technical details of Cinque's account^10^.

**Figure 3:**
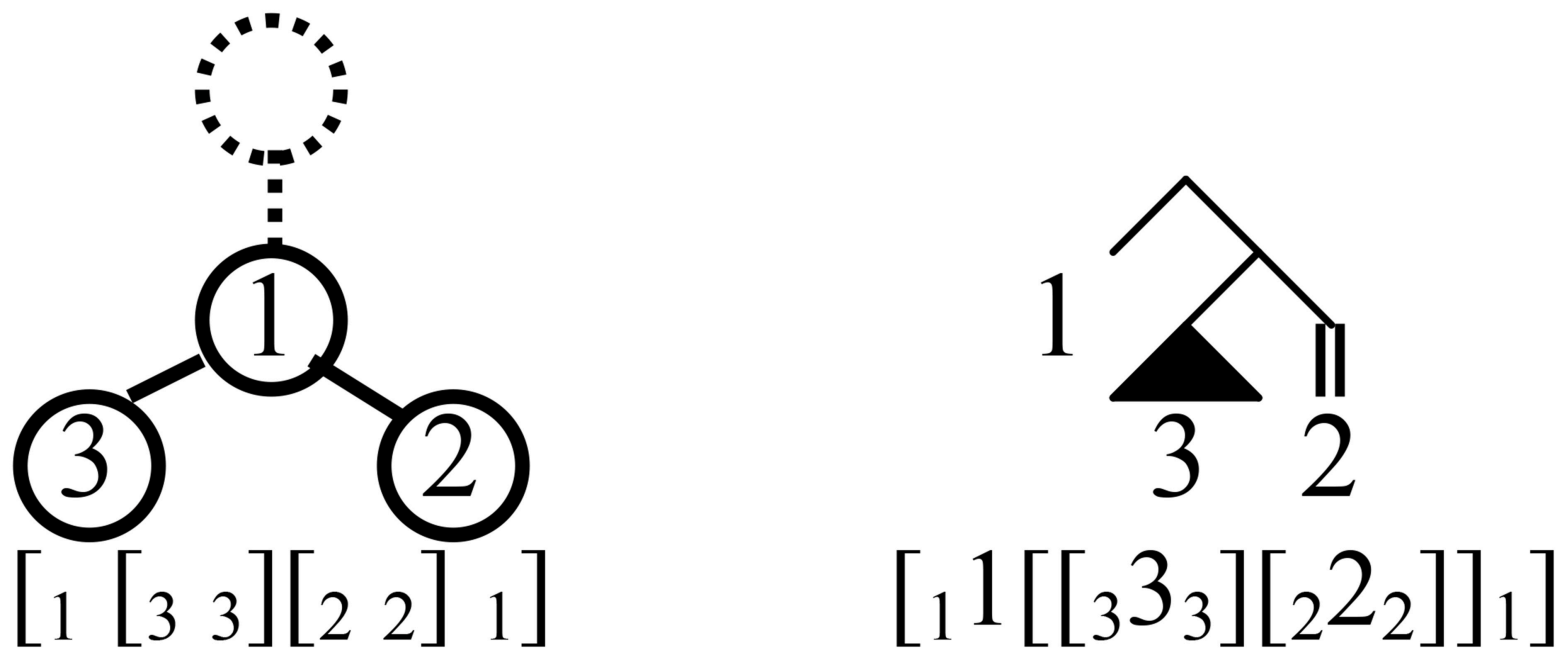
Two bracketed representations of 132 surface order, and corresponding trees. At left is the structure found by reading stack-sorting operations as Brackets; Surface elements are identified with each node (except the topmost, dashed). Linear order is read off top-down along unary-branching paths, and **left-to-right** among sister nodes. In the corresponding binary-branching tree representing its derivation by movement **(right)**, pronounced elements are identified only with terminal nodes.

Setting aside the 321 tree(s) for the moment, the Dyck trees are systematic, loss-less compressions of Cinque's trees, with every subtree that is a right-branching comb in the Cinque tree replaced with a linear tree (see Jayaseelan, 2008) in the Dyck tree. For this correspondence, which amounts to pruning all terminals in the binary tree, the lexical root (e.g., noun in a DP) must not be pruned. Elements from the surface order are associated to each node of the Dyck tree except the highest^11^, with linear order read left-to-right among sister nodes, and top-down along unary-branching paths. For example, for surface order 132, 1 is associated to the sole binary-branching node in its Dyck tree, 3 and 2 to its left and right daughters (Figure 3).

Meanwhile, 321 order, assigned a ternary tree by stack-sorting, has two remnant-movement-avoiding derivations.^12^ In one possible derivation, 3 inverts with 2 immediately after 2 is Merged, then the 32 complex moves past 1 after 1 is Merged. In the other possible derivation, the full base structure is Merged first, then 23 moves to the left, followed by leftward movement of just 3^13^.

A key empirical question is whether 321 orders exhibit two distinct bracketed structures, as binary-branching treatments allow, or only the single, “flat” structure predicted here. The issue is even more acute for 4 elements, as in Universal 20, where there are up to 5 distinct Merge derivations^14^ for 4321 order. Luckily, this (*N Adj Num Dem*) is the most common noun phrase order; future research should illuminate the issue^15^.

### Section summary

Stack-sorting captures a surprising amount of syntactic machinery, normally divided among different modules. In the usual view, an autonomous generative engine builds constituent structures, interpreted at the interfaces by further processes of linearization and composition. In ULTRA, linearization and composition reflect a single procedure. Constituent structure is not primitive, but records the storage and retrieval steps by which stack-sorting assembles the interpretation^16^. This produces a bracketed surface structure, labeled appropriately, largely identical to the bracketed structure in accounts postulating movement (Internal Merge) from a uniform base (formed by External Merge). However, where standard theories countenance multiple derivations for some surface orders (and ambiguous binary-branching structure), the present account assigns unique beyond-binary bracketing. Significantly, there is no role for language-particular features to drive movement. Displacement is handled automatically by stack-sorting, and is in fact its core feature.

## Comparison with existing accounts of universal 20

This section compares the stack-sorting account of Universal 20 to existing Merge-based accounts (Cinque, 2005; Steddy and Samek-Lodovici, 2011; Abels and Neeleman, 2012). I argue that the stack-sorting account is simpler, while avoiding problems that arise in each of these existing alternatives.

### The account of Cinque (2005)

Cinque proposes a cross-linguistically uniform base hierarchy, reflecting a fixed order of External Merge. He proposes that movement (Internal Merge) is uniformly leftward, while the base is right-branching, in line with Kayne's (1994) LCA. He stipulates that remnant movement in the noun phrase is barred: each movement affects the noun, or a constituent containing it. His base structure for the noun phrase is (8).

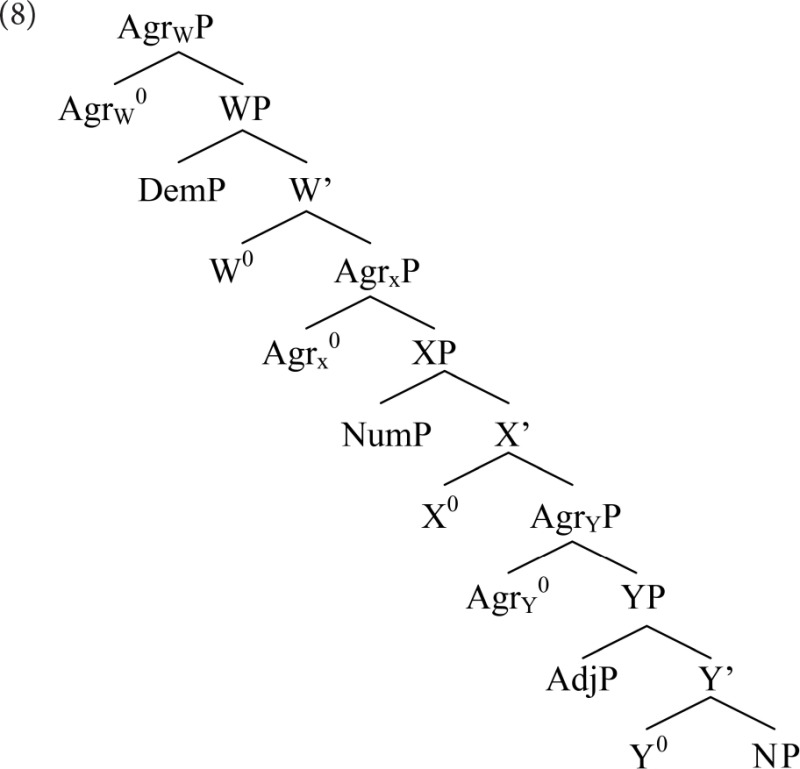

The overt modifiers are specifiers of dedicated functional heads (e.g., X^0^), below agreement phrases providing landing sites for movement. This structure, and his assumptions about movement, derives all and only the attested orders. The English-like order *Dem-Num-Adj-N* surfaces without movement; all other orders involve some sequence of movements of NP, or something containing it.

### The account of Abels and Neeleman (2012)

Abels and Neeleman (2012) modify Cinque's analysis, discarding elements introduced to conform to the LCA (including agreement phrases and dedicated functional heads). They argue that the LCA plays no explanatory role; all that is required is that movement is leftward, and remnant movement is barred. They allow free linearization of sister nodes, utilizing a considerably simpler base structure (9). They omit labels for non-terminal nodes as irrelevant to their analysis (Abels and Neeleman, 2012: 34).

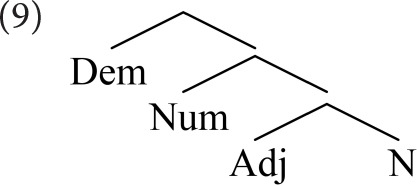

In their theory, eight attested orders can be derived without movement, by varying the linear order of sisters. The remaining attested orders require leftward, non-remnant movement. In principle, their system allows a superset of Cinque's (2005) derivations; some orders can be derived through linearization choices or through movement. However, restricting attention to strictly necessary operations, and supposing that free linearization is simpler than movement, their derivations are generally simpler than Cinque's.

### The account of Steddy and Samek-Lodovici (2011)

Steddy and Samek-Lodovici (2011) offer another variation on Cinque's (2005) analysis. They propose an optimality-theoretic account, retaining Cinque's base structure (8). Linear order is governed by a set of Align-Left constraints (10), one for each overt element.

(10) a. N-L – Align(NP, L, AgrWP, L)
                  Align NP's left edge with AgrWP's left edge.
        b. A-L – Align(AP, L, AgrWP, L)
                  Align AP's left edge with AgrWP's left edge.
        c. NUM-L – Align(NumP, L, AgrWP, L)
                  Align NumP's left edge with AgrWP's left edge.
        d. DEM-L – Align(DemP, L, AgrWP, L)
                  Align DemP's left edge with AgrWP's left edge.
              (From Steddy and Samek-Lodovici, 2011: 450).

These alignment constraints incur a violation for each overt element or trace separating the relevant item from the left edge of the domain, and are variably ranked across languages. Attested orders are optimal candidates under some constraint ranking. The unattested orders are ruled out because they are “harmonic-bounded”: some other candidate incurs fewer higher-ranked violations, under any constraint ranking. Therefore, they can discard the constraints on movement that Cinque (2005) and Abels and Neeleman (2012) adopt. The leftward, non-remnant character of movement instead falls out from alignment principles.

### Problems with existing accounts

Although these accounts differ in details, they share some problematic features. First, all of them capture the word order pattern in three tiers of explanation: (i) a uniform base structure, (ii) syntactic movement, and (iii) principles of linearization. In all three accounts, (i) describes the order of External Merge. Details of (ii) and (iii) vary between the accounts. For Cinque (2005) and Steddy and Samek-Lodovici (2011), all orders except *Dem-Num-Adj-N* involve movement; Abels and Neeleman (2012) require movement for only six attested orders. With respect to linearization, Cinque (2005) utilizes Kayne's (1994) LCA; Abels and Neeleman (2012) have movement uniformly to the left, but base-generated sisters freely linearized on a language-particular basis; Steddy and Samek-Lodovici (2011) have language-particular constraint rankings.

These accounts all require different grammars for different orders. In Cinque (2005) system, features driving particular movements must be learned. The same is true for Abels and Neeleman (2012), with additional learning of order for sister nodes. Steddy and Samek-Lodovici (2011) require learning of the constraint ranking that gives rise to each order. All these accounts face trouble, therefore, with languages permitting freedom of order in the DP; in effect they must allow for underspecified or competing grammars, to capture the different orders.

Finally, all these accounts have some measure of structural or grammatical ambiguity, for some orders. For Cinque (2005), one kind of ambiguity comes about in choosing whether to move a functional category, or the Agreement phrase embedding it; this choice has no overt reflex. Although his theory sharply limits the number and landing site of possible movements, these limitations are somewhat artificial; little substantive would change if we postulated further silent functional layers to host further movements, or allowed multiple specifiers. In the limit, this allows the full range of ambiguous derivations discussed in section Stack-Sorting: Linearization, Displacement, Composition, and Labeled Brackets. Abels and Neeleman's (2012) approach allows this ambiguity among different movement derivations, as well as the derivation of many orders through either movement or reordering of sister nodes. Finally, Steddy and Samek-Lodovici (2011) face a different ambiguity problem: some orders are consistent with multiple constraint rankings (thus, multiple grammars).

### Comparison with the stack-sorting account

The stack-sorting account fares better with respect to these issues. Instead of postulating separate tiers of base, movement, and linearization principles, the relevant machinery is realized in one algorithmic process. The sorting algorithm is universal, eschewing language-particular features to drive movement, order sister nodes, or rank alignment constraints. Such a theory is ideally situated to account for the free word order phenomenon^17^. Furthermore, each order induces a unique sequence of storage and retrieval operations, tracing a unique bracketing. Within domains characterized by neutral word order and a single fseq, there is no spurious structural or grammatical ambiguity, for any word order.

## Universal grammar as universal parser

This section develops the view that stack-sorting can form the basis for an invariant performance mechanism, realizing Universal Grammar as a universal parser. This modifies traditional conclusions about competence and performance, while providing a novel view of what a grammar is.

### Rethinking competence and performance

In generative accounts, a fundamental division exists between competence and performance (Chomsky, 1965). Competence encompasses knowledge of language, conceived of as an abstract computation determining the structural decomposition of infinitely many sentences. Separate performance systems access the competence system's knowledge during real-time processing. In terms of Marr (1982) three-tiered description for information-processing systems, competence corresponds to the highest, computational level, specifying *what* the system is doing, and *why*. Performance corresponds, rather loosely, to the lower, algorithmic level, describing *how* the computation is carried out, step-by-step.

Of course, Marr's hierarchy applies to the information-processing in language, under the present theory as well as any other. However, the division of labor between these components is significantly redrawn here, with much more of the burden of explanation carried by performance^18^. A crucial difference is that in ULTRA, bracketed structure is not within the purview of competence. Instead, such structure arises in the interaction of competence with the stack-sorting algorithm, during real-time parsing. The knowledge ascribed to the competence grammar is simpler, including the innate fseq as a core component^19^. In a way, this aligns with the views of Chomsky's recent work, in which competence is fundamentally oriented for computing interpretations, with externalization “ancillary.”

### A universal parser

A novel claim of ULTRA is that there is a single parser for all languages. This departs from the nearly universal assumption that parsers interpret language-particular grammars. But even within that traditional view, the appeal of universal mechanisms has been recognized.

“The key point to be made, however, is that the search should be a search for universals, even—and perhaps especially—in the processing domain. For it would seem that the strongest parsing theory is one which says that the grammar interpreter itself is a universal mechanism, i.e. that there is one highly constrained grammar interpreter which is the appropriate machine for parsing all natural languages” (Marcus, 1980 p. 11).

The idea that “the parser is the grammar” has a long history; see Phillips (1996, 2003), Kempson et al. (2001), and the articles in Fodor and Fernandez (2015) for recent perspective. Fodor refers to this as the *performance grammar only* (PGO) view.

“PGO theory enters the game with one powerful advantage: there must be psychological mechanisms for speaking and understanding, and simplicity considerations thus put the burden of proof on anyone who would claim that there is more than this” (Fodor, 1978 p. 470).

However, while granting that this entails a simpler theory, Fodor rejects the idea, finding no motivation for movement outside an autonomous grammar (*ibid*., 472). This presupposes that movement is fundamentally difficult for parsing mechanisms (which should prefer phrase-structural mechanisms to transformational ones). However, in ULTRA, displacement is not a complication over more basic mechanisms; displacement *is* the basic mechanism.

### Displacement is not unique to human language

It is often said that displacement is unique to human language, and artificial codes avoid this property^20^. But displacement appears in coding languages, in exactly the same sense that it appears in ULTRA. A simple example illustrates: the order in which users press keys on a calculator is not the order in which the corresponding computations are carried out. In practice, calculators compile input into Reverse Polish Notation for machine use, via Dijkstra's Shunting Yard Algorithm (SYA).

The example is not an idle one; the stack-sorting algorithm (4) is essentially identical to the SYA^21^. Lexical heads (nouns and verbs) are “shunted” directly to interpretation, as numerical constants are in a calculator. Meanwhile the satellites forming their extended projections are stack-sorted according to their relative rank, just like arithmetic operators. In this analogy, cartographic ordering parallels the precedence order of arithmetic operators.

In fact, though the property is little used, the SYA is a sorting protocol; many input orders lead to the same internal calculation. As calculator users, we utilize one input scheme (infix notation), but others would do as well. The standardized input order for calculators has the same status as particular languages with respect to ULTRA: users may fall into narrow ordering habits, but the algorithm automatically processes many other orders.

### Grammaticality and ungrammaticality

One of the central tasks ascribed to grammars is distinguishing grammatical sentences of a language from ungrammatical strings. In ULTRA, knowledge of grammaticality is very different from knowledge of *un*grammaticality. The former kind of knowledge is fundamentally about computing interpretations. But the invariant process interpreting one language's surface order can equally interpret the orders of other languages. From this point of view, there is only one I-language, and a single performance grammar that delivers it. While this conclusion is appealing, an important question remains: where do individual languages come from, with apparently different grammars?

## Possible extensions to a more complete theory of syntax

This section addresses two kinds of problems that follow from interpreting stack-sorting as a performance device. The first concerns reconciling the theory with what is known about real-time language processing; the second concerns extending the model to properties of syntax that are left unexplained. Even discussing these problems in depth, much less justifying any solutions, is beyond the scope of this paper. The intent is merely to sketch the challenges, and indicate directions for further work.

### Reaction vs. prediction: incrementality and rigid word order

With respect to processing, one problem is that this approach seems to be contradicted by strong evidence for word-by-word incrementality in comprehension (especially in the Visual World paradigm; see Tanenhaus and Trueswell, 2006). ULTRA is “pedestrian” in the sense Stabler (1991) cautions against. Within each domain, bottom-up interpretation cannot begin until the lexical root of the fseq is encountered.

One possibility for reconciling ULTRA with incrementality draws on the distinction between reactive processes, such as the stack-sorting procedure, and predictive processing (see Braver et al., 2007; Huettig and Mani, 2016). The idea is that stack-sorting is a reactive mechanism for language perception; this is contrasted with—and necessarily supplemented by—predictive capacities, associated with top-down processing, and production^22^. The latter system alone contains learned, language-particular grammatical knowledge. This proposal echoes other approaches with a two-stage parsing process, such as Frazier and Fodor's (1978) Sausage Machine. ULTRA resembles their Preliminary Phrase Packager (PPP), a fast low-level structure-builder, distinguished from the larger-scale problem-solving of their Sentence Structure Supervisor (SSS). Marcus expresses a similar view, describing a parser as a “fast, ‘stupid’ black box” (Marcus, 1980: 204) producing partial analyses, supplemented with intelligent problem-solving for building large-scale structure.

I suggest that evidence for word-by-word incrementality can be reconciled with the present theory through an interaction between reaction and prediction, exploiting the notion of “hyperactivity” (Momma et al., 2015). The idea is that comprehension can skip ahead, giving the appearance of incrementality, if a lexical root (noun or verb) is provided in advance by prediction. Something like this seems to be true.

“There is growing evidence that comprehenders often build structural positions in their parses before encountering the words in the input that phonologically realize those positions […] To take just one example, in a head-final language such as Japanese it may be necessary for the structure building system to create a position for the head of a phrase before it has completed the arguments and adjuncts that precede the head” (Phillips and Lewis, 2013 p. 19).

A complementary predictive system could help solve two further problems for ULTRA: explaining how production is possible, and why there are distinct languages with different, relatively rigid word orders. The stack-sorting algorithm is a unidirectional parser; there is no trivial way of “reversing the flow” for production. Facing this uncertainty, it would be natural to rely on prediction to supply word order in production^23^. To simplify production, it is helpful for word order to be predictable; in turn, word order tendencies in the linguistic environment can be learned by this system. This suggests a feedback loop, and a plausible route for the emergence and divergence of relatively rigid word orders.

### Primacy vs. recency and the duality of semantics

A number of important syntactic properties remain unexplained. In order to extend the proposal to a remotely adequate theory, these properties must be addressed somehow. These include, first, a cluster of syntactic properties relating to A-bar syntax, and the so-called Duality of Semantics. I suggest that this distinctive kind of syntax relates to an important distinction in short-term memory, between primacy and recency, drawing on Henson's (1998) Start-End Model (SEM)^24^. In Henson's model, primacy and recency are distinct effects, reflecting content-addressable coding of two aspects of serial position.

Recency is naturally associated with stack (last-in, first-out) memory. Primacy, on the other hand, is naturally described by queue (first-in, first-out) memory. Besides optimal order of access, there is another important difference between primacy and recency effects. Put simply, the first element in a sequence remains the first element, no matter how many more elements follow; the primacy signature of a given element is relatively stable over the time scale relevant to parsing. Recency is different: each element in a sequence is a new right edge, suppressing the accessibility to recency-based memory of everything that precedes it. Thus, we expect a kind of “use-it-or-lose-it” pressure within recency memory, but not primacy memory.

Tentatively, I would like to suggest that distinct primacy and recency memory codes underlie the Duality of Semantics, and the division between A-bar and A-syntax. Recency, associated with a stack, is the basis for information-neutral, local permutation, generally characterized by nesting dependencies^25^. Supposing that primacy is crucially involved in non-neutral, A-bar-like syntax suggests an account for a cluster of surprising properties. Most obvious is the association of discourse-information effects with the “left periphery”: the left edge of domains is where we expect primacy memory to play a significant role^26^. An involvement of primacy memory also suggests an analysis of Superiority effects in multiple wh-movement constructions. In Merge-based theories, such constructions (exhibiting crossing dependencies) are problematic, and require stipulative devices like Richards (1997) “Tucking-In” derivations. Thinking of the effects as involving primacy memory suggests a simpler account: ordering of multiple wh-phrases is a matter of first-in, first-out access (queue memory). A final property of this alternative syntactic system that can be rationalized is long-distance movement. Possibly, the availability of long-distance movement for A-bar relations results from the stability of primacy memory, making items encountered in the left periphery accessible for recall later without great difficulty, in contrast to recency memory (which can only support short, local recall). While this is suggestive, addressing the vast literature on A-bar syntax must be left to future research.

### What about recursion?

A final problem looming in the background is recursion. ULTRA operates within syntactic domains characterized by a single fseq. This requires some comment, as recursion is a fundamental property of syntax. For recursion as well, properties of memory, and intervention of a complementary predictive system, might be crucial. Intriguingly, human episodic memory appears to be independently hierarchical in structure, perhaps unlike related animals (Tulving, 1999; Corballis, 2009). In the SEM model, episodic tokens are created for groups, within grouped sequences (Henson, 1998). Linguistic recursion requires some further mechanism for treating the group token corresponding to one sequence as an item token in another sequence.

As discussed in section Comparison with Existing Accounts of Universal 20, in ULTRA, structural ambiguity does not arise without ambiguity of meaning, within single domains. However, structural ambiguity arises inevitably when multiple domains are present, in terms of which domain embeds in another, or where to attach an element that could discharge positions in two distinct domains. This is where the “fast, stupid black box” is helpless, and must call on other resources. One obvious source of help in stitching together multiple domains is a separate predictive system, with access to top-down knowledge of plausible meanings in context. The persistent problem of resolving embedding ambiguities also provides motivation for rigid word order, which sharply reduces attachment possibilities.

An important point is that brackets are defined relative to a particular fseq. Recursive embedding of one domain in another (for example, a nominal as argument of a verb) involves projection of a bracket corresponding to the entire embedded phrase, within the embedding domain^27^. Consider the following example.

(11) The dog chased a ball.

There are three sorting domains here: two nominal projections, embedded in a third, verbal projection (setting aside the possibility that clauses contain two domains, vP and CP phases). Their ULTRA bracketing appears below.

(12) NP1 = [_the_ [_dog dog_] _the_]
        NP2 = [_a_ [_ball ball_] _a_]
          VP = [_NP1_ [_chased chased_] [_NP2 NP2_] _NP1_]

This example illustrates the ambiguity that accompanies embedding. The issue is how to link the nominal phrases to positions in the verb's fseq (i.e., to theta roles). As theta roles are not overtly expressed (case-marking is an unreliable guide), the reactive parser must draw on external means (for example, language-particular ordering habits, or predictions of plausible interpretations).

A final point about recursion returns to the issue of how calculators work, via Dijkstra's Shunting Yard Algorithm. Such computations are recursive. But recursion isn't handled by the parsing algorithm; rather, it arises at the level of interpretation, where partial outputs of arithmetic operations feed into further calculations. A similar conclusion (recursion is semantic, not syntactic) is possible within the present framework, given the similarities between ULTRA and the SYA. Notably, both procedures compile input into Reverse Polish Notation, a so-called concatenative programming language, expressing recursive hierarchical operations unambiguously in serial format.

### Do we even need an algorithm?

I have shown how a particularly simple algorithm captures a range of syntactic phenomena. But the question is, why *this* algorithm? Other sorting procedures are possible in principle, and would lead to different permutation-avoidance profiles. How do we justify selecting stack-sorting as the right procedure for syntactic mapping?

There are three crucial ingredients. The first is the orientation of the system as a parser, mapping sound to meaning. This is not logically necessary; it is simply one of the reasonable choices. The second factor is the linear nature of sound and meaning. This is straightforward for sound sequences, but much less so for interpretations, where it is simply a bold hypothesis. The third ingredient is the choice of stack memory. This can plausibly be tied to the Modality effect: intelligible speech input engenders unusually strong recency effects (Surprenant et al., 1993). It seems a small leap to suppose that the formal stack employed in the algorithm may simply (and crudely) reflect the dominance of recency effects in memory for linguistic material.

So far, stack-sorting has been implemented with an explicit algorithm. That may be unnecessary. Rather than thinking of stack-sorting as a set of explicit instructions, we might reframe it as an *anti-conflict bias* between the accessibility of items in memory, in terms of recency effects, and retrieval for a rigid interpretation sequence. If that is on the right track, it is possible that no novel cognitive machinery had to evolve to explain these effects. What remains is to understand where the ordering of interpretations (the fseq) itself comes from, a matter on which I will not speculate here.

## Conclusion

Summarizing, a simple algorithm (4) maps 213-avoiding word orders to a bottom-up compositional sequence, while mapping 213-containing orders to deviant sequences. While the input and output of the mapping are sequences, hierarchical structure is present: the algorithmic steps realize left and right brackets, almost exactly where standard accounts place them. The account differs from standard accounts in assigning unambiguous bracketing to all orders.

This model improves on existing accounts of word order restrictions, which invoke additional stipulations (e.g., constraints on movement, together with principles of linearization), beyond core syntactic structure-building. In ULTRA, these effects fall out from a single real-time process. In turn, syntactic displacement, long seen as a curious complication, emerges as the fundamental grammatical mechanism. No learning of language-particular properties is required; one grammar interprets many orders.

It should be clear that the system described here is only one part of syntactic cognition. This system builds one extended projection at a time; further mechanisms are required to embed one domain in another. However, that may be a virtue: it is tempting to identify the domains of operation for this architecture with phases, which are thus special for principled reasons.

Moreover, stack-sorting only handles information-neutral structure. This ignores another important component of syntax, so-called discourse-information structure, associated with potentially long-distance A-bar dependencies. This deficiency, too, may be a virtue, suggesting a principled basis for the Duality of Semantics. I speculated that primacy memory plays a central role in these effects, potentially explaining several curious properties (leftness, long distance, and crossing).

Raising our sights, the larger conclusion is that much of the machinery of syntactic cognition might reduce to effects not specific to language. Needless to say, this is just a programmatic sketch; future research will determine whether and how ULTRA's stack-sorting might be integrated into a more complete model of language.

## Author contributions

The author confirms being the sole contributor of this work and approved it for publication.

### Conflict of interest statement

The author declares that the research was conducted in the absence of any commercial or financial relationships that could be construed as a potential conflict of interest.

## Footnotes

^1^This underlies Chomsky's claim that the distinction between External Merge (the base) and Internal Merge (displacement) correlates with the Duality of Semantics: “External Merge correlates with argument structure, internal Merge with edge properties, scopal or discourse-related (new and old information, topic, etc.,)” (Chomsky, 2005, p. 14). However, some neutral word orders require Internal Merge to derive (even allowing free linearization of sister nodes; see Abels and Neeleman, 2012). ULTRA maintains the identification of the base with thematic structure, while rejecting the empirically problematic claim that displacement gives rise to scopal and discourse-information properties.

^2^Schmid and Vogel (2004) report examples of this order in German dialects, but note that focus seems to be involved. Intriguingly, many instances of 213 order are only felicitous under special discourse-information conditions. However, Salzmann has recently described neutral 213 verb cluster orders in Swiss German. I leave this possible counter-example to future investigation.

^3^Verb clusters are an instance of Restructuring, whereby multiple clauses are treated syntactically as monoclausal. Extraposition places the extraposed element in a separate domain. Zwart's observation thus allows us to maintain the generalization that single-domain neutral orders are 213-avoiding.

^4^By focusing on word order, I am also setting aside morphological ordering and features. While I cannot pursue the issue here, there is evidence that morphology obeys similar cross-linguistic restrictions, and there is no reason why the sorting procedure could not apply to sub-word units.

^5^Stack-sorting is usually described as 231-avoiding. However, linguists effectively number their hierarchies backwards, assigning the highest number to the bottom of the hierarchy, the first element interpreted.

^6^In formal language theory terms, stack-sorting is a kind of linear transduction. Linear transduction has largely been ignored as a possible model of grammar, in part because it seemed incapable of describing the hierarchical structure of linguistic expressions. Some researchers (e.g., Marco Kuhlmann and Markus Saers) have recently explored linguistic applications of transduction grammars, in the context of inter-language translation.

^7^Displacement under stack-sorting is limited to word order permutation within a single cycle. Long-distance displacement, such as successive-cyclic wh-movement, requires different mechanisms; see section Primacy vs. recency and the Duality of Semantics.

^8^Stack-sorting is intended as a parsing algorithm. There are standard techniques for extracting bracketed structure from strings with a stack-based parser, such as SR (shift-reduce) parsing. An SR parser has a set of “grammar rules,” specifying licensed surface configurations; when a set of elements on top of the stack match a grammar rule, they may be reduced, replacing them in the stack with the non-terminal symbol from the left-hand side of the rule (e.g., VP, NP on top of the stack may be reduced to S, by the rule S **→** NP VP). A sentence is successfully parsed if fully reduced to the start symbol S; reduction steps realize its phrase-structural analysis. This is quite unlike the stack-sorting procedure, which deploys no grammar rules, nor reduce steps, and applies parsing steps to one element at a time.

^9^The Dyck trees of successive sizes are counted by the Catalan numbers (1, 2, 5, 14, 42, …). These numbers also count permutations avoiding any three-element subsequence.

^10^Technically, in Cinque's theory the dependent modifiers do not label the phrases containing them. Instead, in line with Kayne (1994) Linear Correspondence Axiom, they are phrasal specifiers of silent functional heads. The labeling on the brackets derived instead more closely matches Starke's representations.

^11^This departs from the usual view that words are terminals, with non-terminals representing constituents.

^12^Beyond collapsing ambiguous binary branching to flat, beyond-binary structure, the ternary Dyck tree for 321 order otherwise corresponds to the binary trees as indicated above: prune all terminals in the binary tree, preserving the lexical root (N).

^13^Some might object to extraction from already-moved objects, violating “Freezing.” However, such subextraction is required to derive attested *N-Dem-Adj-Num* (4132) order.

^14^For Cinque (2005), dedicated Agreement Phrases above each modifier-introducing category provide the landing sites of movement. This sharply reduces possible movements. But these AgrPs are technical devices introduced to comply with Kayne's LCA, rather than a central part of his theory. See Abels and Neeleman (2012) for discussion.

^15^Cinque (2005, p. 320) gives the following partial list of languages with this order: Cambodian, Javanese, Karen, Khmu, Palaung, Shan, Thai, Enga, Dagaare, Ewe, Gungbe, Labu and Ponapean, Mao Naga, Selepet, Yoruba, West Greenlandic, Amele, Igbo, Kusaeian, Manam, Fa d'Ambu, Nubi, Kugu Nganhcara, Cabécar, Kunama, and Maori.

^16^The claim that surface structure is an epiphenomenon of processing echoes ideas of Steedman's Combinatory Categorial Grammar (CCG). He argues against viewing “[…] Surface Structure as a level of representation at all, rather than viewing it (as computational linguists tend to) as no more than a trace of the algorithm that delivers the representation that we are really interested in, namely the interpretation.” (Steedman, 2000, p. 3).

^17^What requires explanation, from this point of view, is why languages should settle on distinct, relatively rigid word orders. See section Possible Extensions to a More Complete Theory of Syntax.

^18^Marcus has endorsed this mode of explanation: “[A] theory of parsing should attempt to capture wherever possible the sorts of generalizations that linguistic competence theories capture; there is no reason in principle why these generalizations should not be expressible in processing terms” (Marcus 1980 p. 10).

^19^See Chesi and Moro (2015) for related discussion, and a different perspective.

^20^For example: “These ‘displacement’ properties are one central syntactic respect in which natural languages differ from the symbolic systems devised for one or another purpose, sometimes called ‘languages’ by metaphoric extension (formal languages, programming languages); there are other respects, including semantic differences” (Chomsky, 1995, p. 222).

^21^Thanks to Michael Jarrett for discussion.

^22^The so-called “P-chain” closely identifies prediction and production (see, e.g., Dell and Chang, 2014).

^23^The Dynamic Syntax framework (Kempson et al., 2001) adopts a broadly similar view of production as parasitic on comprehension (thanks to Colin Phillips for discussion).

^24^In discussing memory architectures for language processing, Caplan and Waters (2013) point out that SEM is “reasonably well-established” in the psychological literature as a model of short-term memory, and yet no existing theories of linguistic parsing incorporate it.

^25^Stack-sorting alone can handle some local crossing dependencies. This is surprising, given the usual identification of automata utilizing push-down stacks with context-free grammars, and nesting dependencies. For example, 1,423, 4,132, and 4,231 are attested noun phrase orders (Dem-N-Num-Adj, N-Dem-Adj-Num, and N-Num-Adj-Dem). All three exhibit crossing relations, in that the (selectional) dependency between 4 and 3 crosses the dependency between 2 and 1. In Merge-based accounts, these orders require movement. But as these orders are 213-avoiding, they are stack-sortable.

^26^It may seem suspicious to associate A-bar relations of all kinds to the left periphery; what about wh-in situ constructions? Richards (2010) notes that in Japanese, in situ wh-phrases occur at the left edge of a special prosodic domain, which extends rightward to the complementizer where they take scope.

^27^Psycholinguistic evidence suggests that interpretation of clausal recursion proceeds top-down (Bach et al 1986, Joshi, 1990). Thus, in a recursive structure like [CP1 … [CP2 … [CP3 …]]], the order of interpretation is < CP1, CP2, CP3>. This suggests an intriguing extension of the present theory. Suppose recursive embedding is also parsed by stack-sorting. If the required output of stack-sorting recursive domains is 123-like (top-down) order among cycles, then we predict avoidance of 231-like orders. 231-avoidance is one way of expressing the Final-Over-Final Constraint (FOFC; see Sheehan et al., 2017 for a recent review). Thus, we can explain FOFC effects with this theory, insofar as they obtain over higher-order (recursive) structure. Consider, for example, one robust FOFC effect, the avoidance of V-O-Aux orders across Germanic (and beyond). The nominal is a distinct domain embedded within the clause. The clause itself arguably contains two cycles (vP and CP), with Aux in a higher cycle than V. Then V-O-Aux gives elements of vP, DP, CP, a 231-like order over the top-down embedding hierarchy [CP [vP [DP]]]. If this is on the right track, then 213- and 231-avoidance characterize different levels of structure (bottom-up assembly within local domains, vs. top-down recursion). I leave exploration of this possibility to future work.

## References

[B1] Abels K. Neeleman A. (2012). Linear asymmetries and the LCA. Syntax 15, 25–74. 10.1111/j.1467-9612.2011.00163.x

[B2] Barbiers S. van der Auwera J. Bennis H. Boef E. de Vogelaer G. van der Ham M. (2008). Syntactic Atlas of the Dutch Dialects, Vol. II, Amsterdam: Amsterdam University Press.

[B3] Berwick R. Chomsky N. (2016). Why Only Us: Language and Evolution. Cambridge, MA: MIT Press.

[B4] Braver T. S. Gray J. R. Burgess G. C. (2007). Explaining the many varieties of working memory variation: dual mechanisms of cognitive control, in Variation in Working Memory, eds Conway A. Jarrold C. Kane M. Miyake A. Towse J. (New York, NY: Oxford University Press), 76–106.

[B5] Caplan D. Waters G. (2013). Memory mechanisms supporting syntactic comprehension. Psychon. Bull. Rev. 20, 243–268. 10.3758/s13423-012-0369-9 23319178 PMC3594132

[B6] Chesi C. Moro A. (2015). The subtle dependency between Competence and Performance, in 50 Years Later: Reflections on Chomsky's Aspects, eds Gallego Á. J. Ott D. (Cambridge, MA: MIT Working Papers in Linguistics), 33–46.

[B7] Chomsky N. (1965). Aspects of the Theory of Syntax. Cambridge, MA: MIT Press.

[B8] Chomsky N. (1995). The Minimalist Program. Cambridge, MA: MIT press.

[B9] Chomsky N. (2005). Three factors in language design. Linguistic Inquiry 36, 1–22. 10.1162/0024389052993655

[B10] Cinque G. (1999). Adverbs and functional heads: A cross-linguistic perspective. Oxford: Oxford University Press.

[B11] Cinque G. (2005). Deriving Greenberg's universal 20 and its exceptions. Linguistic Inquiry 36, 315–332. 10.1162/0024389054396917

[B12] Corballis M. C. (2009). Mental time travel and the shaping of language. Exp. Brain Res. 192, 553–560. 10.1007/s00221-008-1491-9 18641975

[B13] Dell G. S. Chang F. (2014). The P-chain: relating sentence production and its disorders to comprehension and acquisition. Phil. Trans. R. Soc. B 369:20120394. 10.1098/rstb.2012.0394 24324238 PMC3866424

[B14] Fodor J. D. (1978). Parsing strategies and constraints on transformations. Linguistic Inquiry 9, 427–473.

[B15] Fodor J. D. Fernandez E. (2015). Special issue on grammars and parsers: toward a unified theory of language knowledge and use. J. Psycholinguist. Res. 44, 1–5. 10.1007/s10936-014-9333-3

[B16] Frazier L. Fodor J. D. (1978). The sausage machine: a new two-stage parsing model. Cognition 6, 291–325. 10.1016/0010-0277(78)90002-1

[B17] Greenberg J. (1963). Some universals of grammar with particular reference to the order of meaningful elements, in Universals of Language, ed Greenberg J. (Cambridge, MA: MIT Press), 73–113.

[B18] Henson R. N. (1998). Short-term memory for serial order: the start-end model. Cogn. Psychol. 36, 73–137. 10.1006/cogp.1998.0685 9721198

[B19] Huettig F. Mani N. (2016). Is prediction necessary to understand language? Probably not. Lang. Cogn. Neurosci. 31, 19–31. 10.1080/23273798.2015.1072223

[B20] Jayaseelan K. A. (2008). Bare phrase structure and specifier-less syntax. Biolinguistics 2, 87–106.

[B21] Joshi A. K. (1990). Processing crossed and nested dependencies: an automaton perspective on the psycholinguistic results. Lang. Cogn. Process 5, 1–27. 10.1080/01690969008402095

[B22] Kayne R. (1994). The Antisymmetry of Syntax. Cambridge, MA: MIT Press.

[B23] Kempson R. Meyer-Viol W. Gabbay D. (2001). Dynamic Syntax. Oxford: Blackwell.

[B24] Knuth D. (1968). The Art of Computer Programming, Vol. 1: Fundamental Algorithms. (Reading, MA: Addison-Wesley).

[B25] Koopman H. (2000). The Syntax of Specifiers and Heads. London: Routledge.

[B26] Marcus M. P. (1980). Theory of Syntactic Recognition for Natural Languages. Cambridge, MA: MIT press.

[B27] Marr D. (1982). Vision: A Computational Investigation into the Human Representation and Processing of Visual Information. New York, NY: W. H. Freeman.

[B28] Momma S. Slevc L. R. Phillips C. (2015). The timing of verb planning in active and passive sentence production, in Poster Presented at the 28th Annual CUNY Conference on Human Sentence Processing (Los Angeles, CA).

[B29] Phillips C. (1996). Order and Structure. Doctoral dissertation, Massachusetts Institute of Technology.

[B30] Phillips C. (2003). Linear order and constituency. Linguistic Inquiry 34, 37–90. 10.1162/002438903763255922

[B31] Phillips C. Lewis S. (2013). Derivational order in syntax: evidence and architectural consequences. Stud. Ling. 6, 11–47.

[B32] Richards N. (1997). What Moves Where When in Which Language? Doctoral dissertation, Massachussetts Institute of Technology.

[B33] Richards N. (2010). Uttering Trees. Cambridge, MA: MIT Press.

[B34] Rizzi L. (1997). The fine structure of the left periphery, in Elements of Grammar: Handbook in Generative Syntax, ed Haegeman L. (Dordrecht: Kluwer), 281–337.

[B35] Schmid T. Vogel R. (2004). Dialectal variation in German 3-verb clusters: a surface oriented OT account. J. Compar. Germanic Ling. 7, 235–274. 10.1023/B:JCOM.0000016639.53619.94

[B36] Sheehan M. Biberauer T. Roberts I. Pesetsky D. Holmberg A. (2017). The Final-Over-Final Condition: A Syntactic Universal, Vol. 76, Cambridge, MA: MIT Press.

[B37] Stabler E. P. Jr. (1991). Avoid the pedestrian's paradox, in Principle-Based Parsing: Computation and Psycholinguistics, eds Berwick R. Abney S. Tenny C. (Dordrecht: Kluwer), 199–237.

[B38] Starke M. (2004).”On the inexistence of specifiers and the nature of heads,” in *The Cartography of Syntactic Structures, Vol. 3: Structures and Beyond*, ed Belletti A. (New York, NY: Oxford University Press), 252–268.

[B39] Steddy S. Samek-Lodovici V. (2011). On the ungrammaticality of remnant movement in the derivation Greenberg's universal 20. Linguistic Inquiry 42, 445–469. 10.1162/LING_a_00053

[B40] Steedman M. (2000). The Syntactic Process. Cambridge, MA: MIT Press.

[B41] Surprenant A. M. Pitt M. A. Crowder R. G. (1993). Auditory recency in immediate memory. Q. J. Exp. Psychol. 46, 193–223. 10.1080/14640749308401044 8316636

[B42] Tanenhaus M. K. Trueswell J. C. (2006). Eye movements and spoken language comprehension, in Handbook of Psycholinguistics, eds Traxler M. Gernsbacher M. A. (New York, NY: Elsevier Academic Press), 863–900.

[B43] Tulving E. (1999). On the uniqueness of episodic memory, in Cognitive Neuroscience of Memory, eds Nilsson L. Markowitsch H. J. (Ashland, OH: Hogrefe and Huber), 11–42.

[B44] Wurmbrand S. (2006). Verb clusters, verb raising, and restructuring, in The Blackwell Companion to Syntax, Vol. V, eds Everaert M. van Riemsdijk H. (Oxford: Blackwell), 227–341.

[B45] Zwart C. J. (2007). Some notes on the origin and distribution of the IPP-effect. Groninger Arbeiten zur Germanistischen Linguistik 45, 77–99.

